# Influenza Virus Like Particles (VLPs): Opportunities for H7N9 Vaccine Development

**DOI:** 10.3390/v12050518

**Published:** 2020-05-08

**Authors:** Peter Pushko, Irina Tretyakova

**Affiliations:** Medigen, Inc., 8420 Gas House Pike, Suite S, Frederick, MD 21701, USA; itretyakova@medigen-usa.com

**Keywords:** H7N9, pandemic influenza A, avian flu, IAV, VLP vaccine

## Abstract

In the midst of the ongoing COVID-19 coronavirus pandemic, influenza virus remains a major threat to public health due to its potential to cause epidemics and pandemics with significant human mortality. Cases of H7N9 human infections emerged in eastern China in 2013 and immediately raised pandemic concerns as historically, pandemics were caused by the introduction of new subtypes into immunologically naïve human populations. Highly pathogenic H7N9 cases with severe disease were reported recently, indicating the continuing public health threat and the need for a prophylactic vaccine. Here we review the development of recombinant influenza virus-like particles (VLPs) as vaccines against H7N9 virus. Several approaches to vaccine development are reviewed including the expression of VLPs in mammalian, plant and insect cell expression systems. Although considerable progress has been achieved, including demonstration of safety and immunogenicity of H7N9 VLPs in the human clinical trials, the remaining challenges need to be addressed. These challenges include improvements to the manufacturing processes, as well as enhancements to immunogenicity in order to elicit protective immunity to multiple variants and subtypes of influenza virus.

## 1. Introduction

In addition to pandemic COVID-19 virus infections, influenza virus remains a major threat to public health and causes significant morbidity and mortality worldwide from annual seasonal epidemics and periodic pandemics [1,2]. According to WHO, seasonal influenza affects 5–10% of the global population annually and results in 3-5 million hospitalizations and about half a million deaths per year. Pandemic influenza A virus (IAV) represents an even greater concern. Due to the rapid mutation rate of human IAVs, antigenically novel viruses are emerging periodically, which are difficult to predict, prevent, or treat [3]. Zoonotic influenza viruses of H5N1, H9N2 and recently, H7N9 and H10N8 subtypes have been identified as potentially pandemic [4,5].

Influenza viruses belong to the *Orthomyxoviridae* family and comprise negative-sense, single stranded, segmented RNA genome. The RNA genome segments are loosely encapsidated by the nucleoprotein into virus particle. There are four types of influenza virus—types A, B, C, and D. Influenza A viruses (IAV) and type B viruses are clinically relevant for humans.

**Figure 1:**
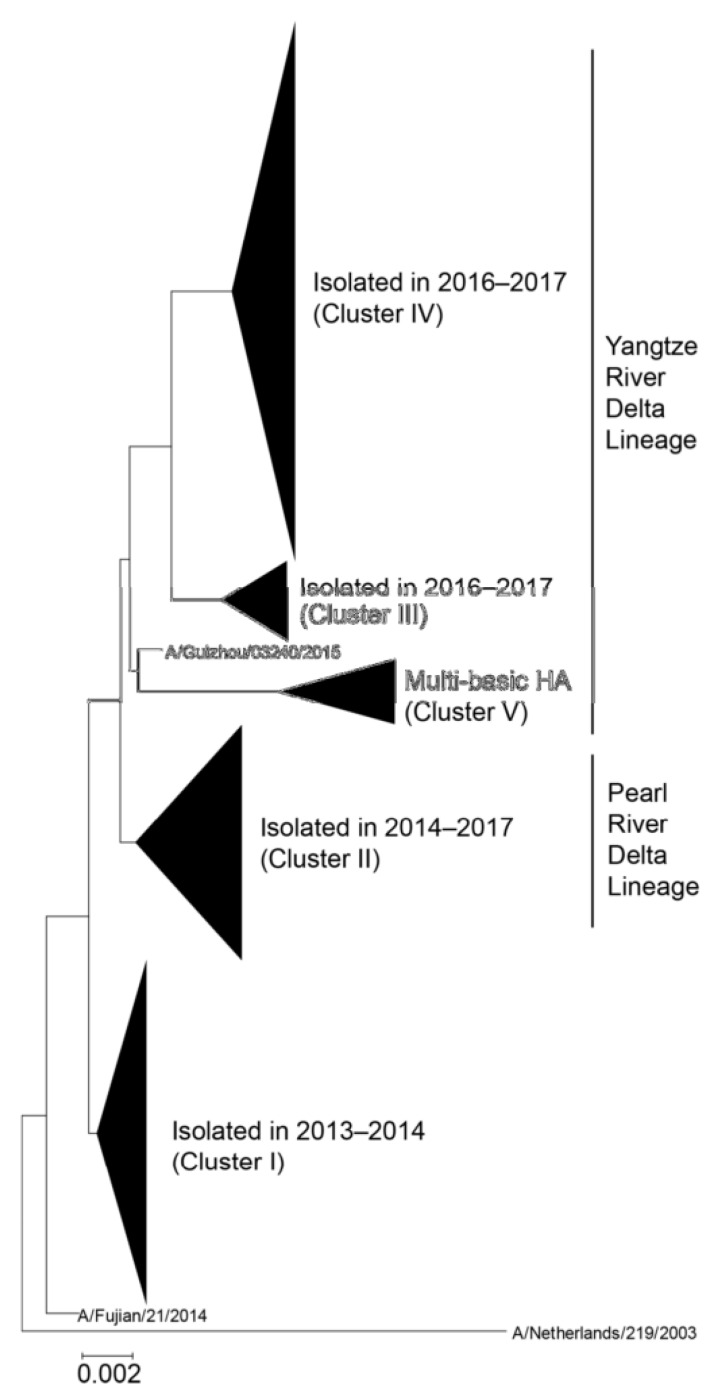
Phylogenetic tree of hemagglutinin (HA) sequences derived from human H7N9 viruses [15]. The evolutionary history was inferred using the neighbor-joining method with Kimura distances. Five major clusters are shown as a collapsed branch. A/Netherlands/219/2003 is defined as an outgroup. The Yangtze River Delta and Pearl River Delta lineages are circulating in China. HPAI H7N9 viruses, which harbor multiple basic amino acids in the HA cleave site, are included in the Yangtze River Delta lineage. Permission: Viruses https://doi.org/10.3390/v11020149.

IAV are further sub-divided based on the antigenic properties of surface glycoproteins into 18 hemagglutinin (HA) and 11 neuraminidase (NA) subtypes. Only a few IAV subtypes have been known to infect humans, while the majority of them are harbored in their natural hosts such as waterfowl, shorebirds, and other species [6]. Cases of H7N9 human infections caused by an avian-origin H7N9 virus emerged in eastern China in March 2013 [7,8]. This novel virus has immediately raised pandemic concerns as historically, pandemics were caused by the introduction of new subtypes into immunologically naïve human populations [9]. Phylogenetic results indicate that novel H7N9 virus was a triple reassortant derived from avian influenza viruses [7]. Since 2013, surveillance of live poultry markets routinely detected H7N9 virus [10]. Human infections with H7N9 virus were associated mainly with the exposure to infected poultry [11] and were identified in many cities in China [12]. Both low pathogenicity avian influenza (LPAI) and high pathogenicity avian influenza (HPAI) H7N9 viruses have been recorded. The first wave of H7N9 was associated with LPAI virus and lasted from March until September 2013. The following four waves occurred annually until 2017 (Figure 1). During the fifth wave in the 2016/17 season, the emergence of HPAI H7N9 viruses was detected. After no reported human cases of HPAI H7N9 for over a year, another HPAI H7N9 case with severe disease was reported in mainland China in late March 2019, indicating the continuing public health threat from the H7N9 subtype [13]. HPAI subtype H5 and H7 proteins contain multiple basic amino acid cleavage sites between HA1 and HA2 domains within HA proteins, which can be cleaved by furin-like proteases [14] in many host cells and organs that can lead to the efficient spread of the virus and severe disease in humans. In contrast, HA of LPAI viruses does not have the furin cleavage site.

A fatality rate of up to 38% was reported for H7N9 viruses [16], which highlights the need for a safe and effective vaccine [17]. Several candidate H7N9 vaccine viruses have been prepared and listed by WHO (Table 1). These candidate vaccine viruses are available to vaccine developers for the preparation of H7N9 vaccine in the case of a pandemic. The majority of current vaccine manufacturers prepare vaccines either as split subvirions or live-attenuated viruses, and they are mostly dependent on fertilized chicken eggs as production “bioreactors”. This technology is unlikely to meet the vaccine production demand during the rapid pandemic spread [18]. Scalability issues (one vaccine dose/egg), the relatively long 6-month time period from strain isolation to final dose formulation and the requirement of biosecurity facilities for HPAI are the major obstacles for egg-based production [19]. In addition, IAV can acquire adaptive mutations when grown in eggs, which can interfere with the vaccine performance and efficacy. According to the action plan published by WHO in 2006, more than 2.34 billion monovalent vaccine doses will be needed in the case of a global pandemic, which justifies the development of novel technologies capable of supporting surge demand for pandemic influenza vaccine within a short period of time [20].

## 2. Recombinant Influenza Virus-Like Particles (VLPs) as Vaccines

Recently, several new platform technologies including recombinant VLPs have been developed in order to facilitate the scale-up of vaccine production including overcoming the drawbacks of the egg-based vaccine production method. The first recombinant influenza vaccine FluBlok based on HA antigen has received FDA approval [22]. The surface envelope glycoproteins such as influenza HA and NA generally are viewed as the primary targets for vaccine development. HA currently represents the major target for vaccine development including approved and experimental vaccines [22]. Recombinant HA-based vaccines have been shown to be efficacious against influenza including H7N9 [17,23,24,25,26,27,28,29,30,31]. Experimental NA-based vaccines have been reported [32,33]. The induction of immune responses against the surface envelope proteins has advantages, because virus-neutralizing antibody response to the viral envelope proteins can prevent early steps of viral infection. Human monoclonal antibodies targeting the HA glycoprotein can neutralize H7N9 influenza virus [34]. Importantly, the neutralizing antibodies protected against A/Shanghai/2/2013 (H7N9) virus challenge [35].

**Figure 2:**
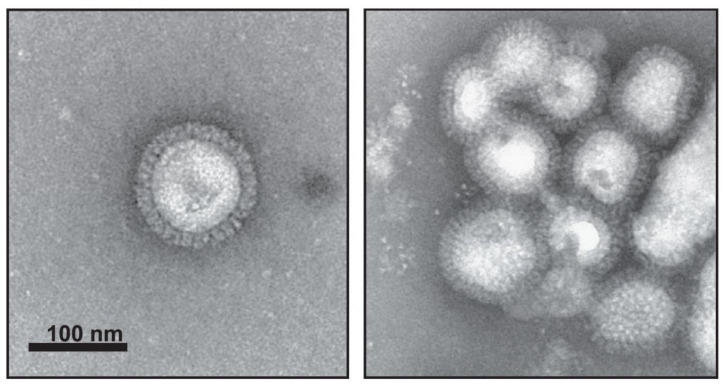
Electron microscopy images of influenza VLPs prepared in Sf9 cells using baculovirus expression system. Recombinant VLPs contain three subtypes (H5, H7, H9), morphologically and biochemically are similar to the wild type influenza virus, and can be found as individual particles (left) or groups of particles (right). Characterization of triple-subtype VLPs was done by negative staining transmission electron microscopy. (Adapted from [50], Copyright 2013, with permission from Elsevier, license 4798470004544).

The novel recombinant VLP vaccine platforms included VLPs prepared in mammalian, insect, and plant expression systems. Recombinant VLPs are morphologically and biochemically similar to the wild type influenza virus (Figure 2); however, they lack viral genetic material and are unable to replicate and cause infection. Table 2 shows examples of various VLPs prepared from H7N9 virus antigens. Cell culture-based production of VLPs can potentially overcome limitations of classic influenza vaccines [36].

As VLPs closely resemble viruses, they contain immunological epitopes in the natural conformation, are highly immunogenic, but they are non-infectious because they are prepared in the absence of viral genomic RNA segments. Each VLP represents a repetitive and highly organized molecular array of antigens displayed on the VLP surface. VLPs have been shown to be better immunogens than subunit vaccines because of their self-adjuvant properties [51]. The antigens present in VLP bind pattern recognition receptors on innate immune effector cells and trigger the innate immunity of host cells [52,53]. The molecular arrays displayed on VLP’s surface are also potent inducers of Type 2 T-independent B-cell response [54]. Cryo-electron microscopy of VLPs from 1918 pandemic influenza virus demonstrated a uniform distribution of HA molecules on the surface of VLPs along with prefusion state confirmation [55]. Two types of spike on the surface of virus are formed by trimers of HA and tetramers of NA. It has been estimated that a spherical influenza virion of average diameter 120 nm has ~375 spikes [56].

## 3. Recombinant Platforms for Expression of H7N9 VLPs

The recombinant platforms using either mammalian, insect, or plant cells have many potential advantages including scalability and intrinsic safety, especially for HPAI viruses such as H7N9 (Table 2). Mammalian cells, such as MDCK, Vero and PERC.C6 cell lines have been extensively studied and applied to influenza vaccine production. The use of VLPs expressed from mammalian cell platform has advantages for VLP production such as flexibility and a similar glycosylation pattern to the human virus. In some reports, VLPs expressed from HEK293T cells containing HA and NA proteins on their surface with a HA:NA ratio of 1:1, which is higher than natural influenza viruses, where the HA:NA ratio is 4:1 [57]. VLPs isolated from HEK293SF cells were also characterized by nano-LC-MS/MS analysis to identify the protein cargo. Nucleolin was the most abundant cellular protein present in VLPs, in addition to the recombinant influenza VLP proteins [58]. However, production of VLPs in mammalian cell expression system is difficult to scale up to the commercial scale, especially when using the transient transfection method. This limitation can be addressed by preparing stable cell line of corresponding genes. For example, a stable cell line expressing H1 and N1 genes under the cumate inducible promoter was generated to create 293HA-NA cells. Plasmid with either Gag or M1 gene was transiently transfected in 293HA-NA cells and VLP yield was compared with Gag or M1 protein. 293HA-NA cells produced seven times more HA and NA with Gag plasmid transfected cells than its counterpart matrix M1-encoding plasmid [59]. Gag VLPs produced by this approach elicited strong antibody response and provided complete protection against the homologous virus strain. Optimization of cell density at the time of induction in inducible stable HEK293 cell lines also increased VLP yield by five-fold [60].

Insect cells such as Sf9 can be maintained in serum-free suspension cultures and have been often used to prepare recombinant VLPs including H7N9. When influenza VLPs were compared between insect Sf9 cells and mammalian HEK293 suspension cells, Thompson et al. found that Sf9 cells produce approximately 35 times more VLP than HEK293 cells [61]. VLPs produced from Sf9 cells also showed higher HA titer and more homogenous VLPs than HEK293 cells derived VLPs [61]. As expected, the high titer of baculovirus vector was also present in VLPs prepared in Sf9 cells. However, VLPs generated in HEK293 cells contained extracellular vesicles, such as exosomes and microvesicles. VLPs produced from Sf9 insect cells may have a safety advantage over other expression system because insect cells are unlikely to contain human pathogens, and baculovirus vector cannot replicate in human cells. VLPs produced from insect cells show higher yields as compared to mammalian cell-produced VLPs, which is beneficial for commercial production. In the above study, Sf9 cells produced up to 35 times higher yield than the HEK293 cells’ expression system [61]. In addition, VLPs had higher HA activity and they were more homogenous in morphology and size than mammalian cells produced VLPs [61]. Sf9 cells are usually maintained in a serum free, animal product-free medium. Some of the chemical compounds known as M2 inhibitors, such as amantadine-enhanced expression of HA protein in VLPs by seven-fold in Sf9 expression system [62]. However, the challenge for VLPs expressed using baculovirus expression system is that it is difficult to separate VLPs from the baculovirus expression vector during vaccine manufacturing.

H7N9 VLPs have been prepared from the full-length HA, NA, and matrix M1 genes in Sf9 cells. The H7 HA was derived from A/Shanghai/2/2013 (H7N9) virus, while both NA and M1 proteins were derived from A/Puerto Rico/8/1934 (H1N1) virus, a standard donor virus that is often used for preparation of influenza reassortant viruses used in human influenza vaccine production [63]. VLPs have been isolated from Sf9 cells and their morphology resembling native H7N9 virus envelope was confirmed.

In another study, recombinant H7N9 VLP vaccine was prepared from HA and NA derived from the A/Anhui/1/2013 (H7N9) virus and the matrix M1 protein derived from the H5N1 virus (A/Indonesia/05/2005) [42]. As control vaccines, VLPs were prepared from A/chicken/Jalisco/CPA1/2012 (H7N3) and A/Indonesia/05/2005 (H5N1) viruses. The H7N9 VLPs elicited hemagglutination-inhibition (HAI) antibody response against the homologous H7N9 virus, cross-reactive HAI against the heterologous H7N3 virus, and three- to four-fold higher HAI response if VLPs were administered with ISCOMATRIX™ saponin-based adjuvant. Similarly, all doses of H7N9 VLP elicited anti-NA antibody, with three- to four-fold higher responses measured in the corresponding ISCOMATRIX subgroups. A lethal murine wild-type A/Anhui/1/2013 (H7N9) challenge demonstrated 100% survival of all animals receiving H7N9 or H7N3 VLP vaccines, versus 0% survival in A(H5N1) vaccine and placebo groups [42].

Another study reported a VLP from Sf9 cells that consisted of HA, NA and M1 proteins derived from the human isolate A/Taiwan/S02076/2013(H7N9) as a potential vaccine. In animal experiments, BALB/c mice and specific-pathogen-free chickens receiving the VLP immunization developed HAI serum titer and antibodies against NA and M1 proteins [45].

The immunogenicity and protective efficacy of a H7N9 VLP vaccine were evaluated in the ferret challenge model considered the most appropriate animal model for influenza [31]. Purified recombinant H7N9 VLPs morphologically resembled influenza virions and elicited high-titer serum HAI and neutralizing antibodies to A/Anhui/1/2013 (H7N9) virus. H7N9 VLP-immunized ferrets were challenged with homologous virus. After challenge, VLP-vaccinated animals displayed reductions in fever, weight loss, and virus shedding as compared to the same parameters in the unimmunized control ferrets. H7N9 VLP was also effective in protecting against lung and tracheal infection. The addition of either ISCOMATRIX or Matrix-M1 adjuvant further improved immunogenicity and protection of the VLP vaccine against H7N9 virus [31].

Another recombinant influenza vaccine was developed by expressing H7 from H7N9 (A/Shanghai/2/2013) on the surface of recombinant baculovirus. Although this approach would not result in a typical influenza VLP morphology, the spatial conformation of H7 in the resulting vaccine is expected to be similar to that in an influenza virion or in a cognate VLP. Mice were immunized twice, either intranasally or subcutaneously, with the vaccine. The immunogenicity and cross-protective efficacy of the vaccine were evaluated against H7N9 or H7N7 subtype challenges. The authors concluded that intranasal administration of H7 protein expressed on the baculovirus envelope can be an alternative way to prime the immune system against influenza infection during a pandemic situation [64]. Recently, a baculovirus vaccine expressing the HA of H7N9 strain A/Chicken/Jiaxing/148/2014 was prepared [65]. The recombinant baculovirus-HA generated in this study showed favorable growth characteristics in insect cells, good safety profile, and induced high-level hemagglutination inhibition antibody titer. Moreover, this vaccine demonstrated better efficacy than inactivated whole-virus vaccine JX148, provided complete protection of chickens against challenge with HPAI H7N9 virus, and effectively inhibited viral shedding.

Plant-based VLP vaccines can be produced from *Agrobacterium*-mediated transient expression of influenza HA proteins in *Nicotiana benthamiana*. Plant-made H1 or H5 VLPs mimicked the structure of influenza virions to some extent, they are immunogenic and elicit both humoral and cellular responses [66,67]. Each HA-only particle contains 30–50 homo-trimer HA incorporated into lipid bilayer envelope of plant cell origin [68]. Plant-based recombinant VLP vaccines elicited humoral and cellular responses and protected against challenges. For example, a plant-derived VLP vaccine based on the HA of influenza H7N9 A/Hangzhou/1/2013 was prepared, with no other influenza proteins included in the vaccine. The immunogenicity of such H7-only VLP vaccine was assessed in mice and ferrets after one or two intramuscular dose(s) with, and without, adjuvant (alum or GLA-SE™). In ferrets, H7-specific cell-mediated immunity was also evaluated. The mice and ferrets were challenged with H7N9 A/Anhui/1/2013 influenza virus. A single immunization with the adjuvanted vaccine elicited a strong humoral response and protected mice against an otherwise lethal challenge. Two doses of unadjuvanted vaccine significantly increased humoral response and resulted in 100% protection with significant reduction in clinical signs, leading to nearly asymptomatic infections. In ferrets, a single immunization with the alum-adjuvanted H7 VLP vaccine induced strong humoral and CMI responses with antigen-specific activation of CD3(+) T cells. This plant-made H7 vaccine therefore induced protective responses after either one adjuvanted or two unadjuvanted doses [39]. Potentially, the plant-based transient expression system can allow the production of VLP structures, which contain HA only, without other influenza proteins.

The recombinant HA-only H7 vaccine against H7N9 virus has been also prepared in insect cells [30]. The study has shown that purified HA formed rosette-shaped nanoparticles of approximately 30–50 nm in diameter [69]. Similarly, subviral particles containing the full-length H7 rHA (A/Anhui/1/2013 (H7N9) were isolated from Sf9 cells treated with mild detergent [38]. Similarly to a previous study [30], purified rHA formed particulate structures; the particles were approximately 20 nm in diameter, exhibited hemagglutination activity, and consisted of approximately 3–4 trimers of the full-length HA molecules.

## 4. Considerations for Immune Responses and Protection

As noted above, immunization with VLPs have shown promising results in protecting from influenza infections with H7N9 [27,70,71] (Table 2). Potentially, antibody induced by VLPs can be more effective than those of subunit vaccines containing recombinant protein antigens [72]. In addition, experimental VLP vaccines generally show higher protective rates to high-risk groups such as children and the elderly [26]. However, the full spectrum of factors affecting immunogenicity of VLPs requires additional studies. For example, it has been shown that amino acid residues of HA that are related to receptor specificity can affect the protective efficacy of H5N1 and H7N9 vaccines in mice [44]. H7N9 VLP vaccine that contained L226 (mammalian specificity) and G228 (avian specificity) in HA showed better immunogenicity and protection efficacy than VLP containing HA with either L226 + S228 or Q226 + S228. This observation indicated that specific HA residues could enhance a vaccine’s protection efficacy and HA glycoproteins with both avian-type and human-type receptor specificities may produce better pandemic influenza vaccines for humans [44].

While inactivated vaccines have been shown to induce predominantly systemic humoral response, VLP vaccines stimulate both humoral and cellular immune responses. H7N9 VLPs secreted from 293T cells triggered both humoral and cellular immune responses in mice. This vaccine produced higher levels and antibody and isotypes of IgG, as well as cross-reactive HAI titer against heterologous H1N1 and H1N3 subtypes [43].

The presence of NA and potentially other IAV proteins in VLP could be beneficial. The NA was demonstrated to protect host from influenza infection [73]. Mice immunized with NA VLPs (without HA) were protected against lethal challenge of homologous A/PR/8/34 virus without any weight loss [74].

Furthermore, both the exterior and interior of VLPs can be altered to enhance their stability and immunogenicity. Various adjuvants such as alum, CpG DNA, monophosphoryl lipid A (MPL), poly IC, gardiquimod, cholera toxin (CT) can be encapsulated into VLPs by exogenous and endogenous methods [75,76]. These adjuvanted VLPs show higher levels of antibodies in both sera and mucosa. In another study, adjuvants that stimulate TLR3 or NLPR3 pathways showed higher efficiency of influenza VLP vaccine in aged group of mice [77]. The CpG-adjuvanted intranasal immunization with an egg-derived split H7N9 vaccine offered a high level of protection against H7N9 infection in mice [78].

## 5. Human Clinical Trials with H7N9 VLP Vaccines

Two phase I clinical trials of experimental H7N9 VLP vaccines with, and without, adjuvant have been completed, NCT01897701 and NCT02078674. The first study enrolled 284 adults (≥18 years of age) in a randomized, observer-blinded, placebo-controlled clinical trial [79]. Interestingly enough, significant increases in N9 neuraminidase-inhibiting antibodies occurred in up to 71.9% of recipients of the vaccine without adjuvant, 92.0% of recipients of vaccine with 30 units of adjuvant, and 97.2% of recipients of vaccine with 60 units of adjuvant. Remarkably, the vaccines that were studied were released for human use within 3 months after the availability of the HA and NA sequences. This illustrates the possibility of rapid response to the public health emergency situations, such as in the case of pandemic influenza outbreaks, as well as other health emergencies such as the ongoing COVID-19 coronavirus pandemic.

In another previously reported phase I clinical trial, subjects vaccinated with two doses of an unadjuvanted H7N9 VLP vaccine responded poorly (15.6% seroconversion rates with 45 μg HA dose). In contrast, 80.6% of subjects receiving H7N9 VLP vaccine (5μg HA) with ISCOMATRIX adjuvant developed HAI responses [80]. The results suggest that adjuvants can be important component for the development of safe and effective H7N9 human vaccines [81].

Plant-derived quadrivalent seasonal VLP vaccine has been evaluated and elicited cross-reactive antibody and T cell response in healthy adults [82]. Although H7N9 antigens have not been included in this study, the confirmed safety profile of VLP in humans suggests an attractive alternative manufacturing method for producing effective and HA-strain matching influenza vaccines.

## 6. Broadly Protective Influenza VLP Vaccines

Current annual vaccines (inactivated, live attenuated, and recombinant subunit) protect from circulating, antigenically matching IAV and influenza B virus strains. Broad protection is difficult to achieve because of the frequent emergence of new strains [83]. Therefore, current approved and experimental vaccines have limited protection against antigenically mismatched variants and newly emerging viral strains. For new strains, antigenically matching new vaccines have to be developed within short periods of time. Therefore, vaccines are needed that can protect from an antigenically divergent strain, especially from potentially pandemic viruses with a new HA subtype. In addition to H7N9 virus described above, multiple other viruses with pandemic potential, including H5N1, H9N2, and H10N8 subtypes, that have caused human infections in the past, continue to circulate in birds and other animals. Therefore, vaccines capable of protecting against multiple potentially pandemic influenza strains would be advantageous for pandemic preparedness and public health.

One way to achieve protection against multiple strains and increase the breadth of immune response afforded by VLPs can be a blended formulation of monovalent vaccines. A vaccine was prepared by mixing VLPs that display H1, H3, H5 or H7 HA molecules. Mice vaccinated with these VLPs intranasally showed significant protection (94% aggregate survival following vaccination) against 1918 H1, 1957 H2, and avian H5, H6, H7, H10 and H11 HA subtypes [41]. These experiments suggest a promising and practical strategy for developing a broadly protective influenza vaccine.

**Figure 3:**
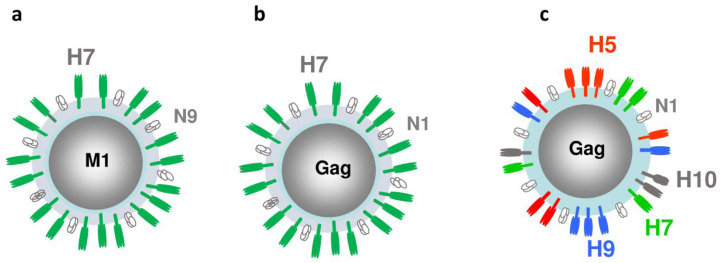
Structural proteins in mono- and quadri-subtype VLPs. (**a**) Mono-subtype H7N9 VLP with H7, N9 and M1 genes; (**b**) Mono-subtype chimeric H7N1 VLP with H7, N1, and Gag genes; (**c**) Quadri-subtype VLPs co-localizing H5, H7, H9 and H10 subtypes along with N1 and Gag within a VLP.

Potentially, HA molecules from different subtypes can be co-localized within recombinant VLP envelope for broader immune coverage. A multi-subtype, mosaic VLP design containing three or four subtypes of the full-length rHA within the envelope has also been described [50,71]. A triple-subtype VLP contained HA proteins from the potentially pandemic H5N1, H7N2, and H9N2 subtypes [50]. A recombinant baculovirus vector was prepared to co-express the H5, H7, and H9 genes from A/Viet Nam/1203/2004 (H5N1), A/New York/107/2003 (H7N2) and A/Hong Kong/33982/2009 (H9N2) viruses, respectively, as well as NA and M1 genes from A/Puerto Rico/8/1934 (H1N1) virus. VLPs were prepared in Sf9 cells, and the immunogenicity and efficacy of the resulting H5/H7/H9 VLPs were evaluated in a ferret animal model following intranasal (i.n.) vaccination. We showed that i.n. vaccination with the H5/H7/H9 triple-subtype VLP induced immune responses and protected ferrets from experimental challenges with three subtypes of avian influenza viruses [50]. We also prepared triple-HA mosaic VLPs that co-localized A/Vietnam/1203/2004 (H5N1), A/Hong Kong/33982/2009 (H9N2) and A/Shanghai/2/2013 (H7N9) rHA proteins, as well as quadri-HA VLPs (Figure 3).

By using Group antigen (Gag) derived from bovine retrovirus as a VLP core in place of M1 protein, quadri-subtype VLPs were prepared, which co-expressed within the VLP the four HA subtypes derived from avian-origin H5N1, H7N9, H9N2 and H10N8 viruses. VLPs showed hemagglutination and neuraminidase activities and reacted with specific antisera [84]. Quadri-subtype vaccine elicited serum antibody in ferrets to the homologous H5, H7, H9, and H10 antigens. Antiserum was also evaluated for cross-reaction with multiple clades of H5N1 virus, and cross-reactivity has been confirmed. The level of immune response suggests protection against multiple influenza subtypes, which was experimentally confirmed by challenge with H10 IAV. Ferrets were protected from challenge with H10 virus [85]. Overall, such multi-subtype, mosaic VLPs that co-localize distinct HA subtypes in the envelope showed broader protection range against different influenza viruses [47,85,86]. Multi-subtype mosaic VLPs combine advantages of conserved HA epitope and blended VLPs, as VLPs contain both subtype-specific head epitopes and the conserved stem epitopes.

Recently, a VLP preparation consisting of retroviral Gag-VLPs pseudo-typed with the HA was expressed using the novel *Trichoplusia ni* (*T.ni*)-derived insect cell line *Tnms*42 and tested successfully to assess the sole contribution of anti-HA immunity in limiting post-influenza secondary *Staphylococcus aureus* bacterial infection, morbidity and mortality in a situation of a vaccine match and mismatch [87]. The results demonstrate that matched anti-HA immunity elicited by a VLP preparation may suffice to prevent morbidity and mortality caused by lethal secondary bacterial infection.

It should also be noted that cross-reactive antibodies to the H7N9 virus were also induced by recombinant viral vectors, such as Newcastle disease virus (NDV) [88,89,90] and parainfluenza virus PIV5 [91].

## 7. Expression of H7N9 Influenza Epitopes Using VLP Carriers

**Figure 4:**
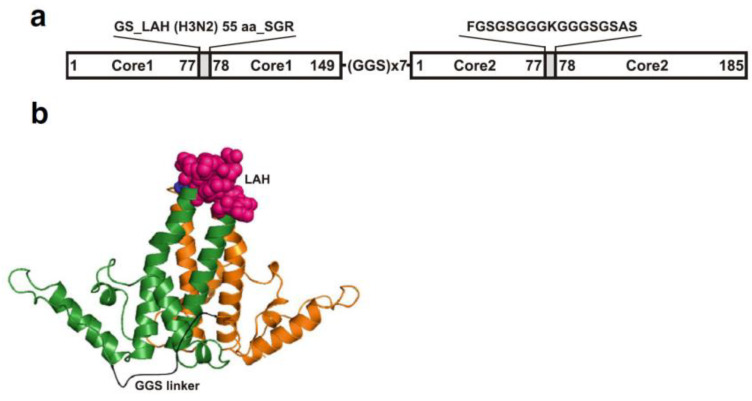
An example of expression of influenza epitope using chimeric VLP based on recombinant LAH3-HBc gene. Design (**a**) and cartoon (**b**) of the LAH3-HBc dimer. Individual hepatitis B virus core protein (HBc) monomers are shown in green and orange. The first major immunodominant region (MIR) contains the LAH domain (the 55 amino acid long influenza H3N2 virus (A/Hong Kong/1/1968, Accession No. AAK51718) HA stalk domain, corresponding to HA amino acids 420–474, shown as pink spheres) while the second MIR contains lysine linker (blue spheres)). The model was created using PyMOL version 1.7rc1. The tandem core dimer is based on the structure from PDB-1QGT, with the linker shown in black.

The emerging strains potentially can be also targeted by “universal” vaccines consisting of conserved viral proteins or epitopes, such as stem region of HA, or extracellular domain M2e of the ion channel protein M2, which are both considered capable of inducing cross-reactive immune responses. Targeting the stem region of HA for antibody production could be a promising approach to generate a broadly protective influenza vaccine. The immune response can be elicited against both the head and stem region of HA protein. However, because of the constantly changing nature of the head region due to the antigenic drift, new vaccine candidates need to be updated frequently. Since the stem region is evolutionally more stable and more conserved among different influenza strains, vaccine candidates targeting the stem region might not need to be updated every year and could induce a broad range of protective immunity against different influenza strains [92,93]. However, the stem region is less immunogenic in the virus. Therefore, chimeric VLP approaches have been used to elicit antibodies to the stem region of HA. The long alpha-helix (LAH) region located in influenza virus hemagglutinin (HA) shows conservation among different influenza A strains, and could be used as a candidate target for influenza vaccines. The hepatitis B virus core protein (HBc) was used as a carrier for heterologous LAH epitopes to elicit effective immune responses [94]. The spatial conformation of LAH epitope cloned in the major immunodominant region (MIR) of the HBc molecule and expressed in yeast is shown on Figure 4. The LAH region of the H7N9 influenza virus was inserted into the HBc to prepare chimeric LAH-HBc protein, which was capable of self-assembly into VLPs in *E. coli* expression system [95]. Intranasal immunization of the LAH-HBc VLP in combination with chitosan adjuvant or CTB∗ adjuvant in mice induced both humoral and cellular immune responses effectively and conferred complete protection against lethal challenge with homologous H7N9 virus or heterologous H3N2 virus, as well as partial protection against lethal challenge of heterologous H1N1 virus. These results provide a proof of concept for LAH-HBc VLP vaccine that can be rapidly produced and potentially can serve as an antigen against a future influenza pandemic [95].

In another study, the globular head domain (HA1-2, aa 62–284) of the protective H7 HA was fused to the potent TLR5 ligand, *Salmonella typhimurium* flagellin (FliC) [96]. The resulting fusion protein, HA1-2-fliC, which apparently did not form VLPs, was efficiently expressed in *E. coli*, retained the native HA and fliC conformations, and was highly immunogenic in mice by intraperitoneal vaccination. Furthermore, highly immunogenic influenza VLPs have been prepared via the overexpression of four viral proteins, HA, NA, M1, and M2, using M2 fusion with the FliC [97]. The chimeric H5N1 VLPs were further combined with the molecular adjuvant, granulocyte-macrophage colony-stimulating factor (GM-CSF), FliC, or a GM-CSF/FliC [98]. All three forms of the chimeric H5N1 VLPs elicited protective immunity against live virus. Next, the GM-CSF/FliC H5N1 VLPs were obtained to include H7 or H1H7 antigens for developing multi-subtype influenza vaccines [98].

The chimeric norovirus VLPs, P particles, were used to express the trivalent HA2:90-105 epitopes derived from H1, H3 and B subtypes, with 24 copies in total, on the surface loops [99].

Finally, trimeric H7 transiently expressed in *N. benthamiana* was conjugated successfully onto the surface of nanodiamond particles [100]. After two or three immunizations in mice, the mixture of trimeric H7 protein and nanodiamond elicited statistically significant stronger H7-specific-IgG response demonstrated by higher amounts of H7N9-specific IgG.

Other universal influenza vaccine approaches applicable to H7N9 were based on ion channel protein M2. Extracellular part of M2 protein, ~23 aa residues, is highly conserved among human IAV strains, suggesting its potential utility as a broadly protective immunogen for the development of broadly protective influenza vaccines. Tandem repeats of heterologous M2e sequences (M2e5x) derived from human, swine, and avian origin influenza A viruses were expressed on influenza VLPs in a membrane-anchored form. Immunization of mice with M2e5x VLPs induced antibodies that were cross-reactive to antigenically distinct influenza A viruses and conferred cross-protection [101]. Furthermore, the M2e5x VLPs demonstrated a clear advantage in inducing IgG2a isotype antibodies, T cell responses, plasma cells and germinal center B cells as well as in conferring cross protection [102]. These studies paved the way to a novel vaccination strategy by enhancing the cross protective efficacy of live attenuated influenza virus vaccines by supplemented vaccination with M2e5x VLPs [103].

High immunogenicity in mice, even in the absence of adjuvants, was demonstrated by the M2e displayed on VLPs of *Macrobrachium rosenbergii* nodavirus [104,105]. This novel vaccine candidate was tested by the H1N1 and H3N2 challenge in mice, and potentially, it can be universal and applicable to H7N9 virus. In another example, the M2e proteins, as well as NA, were expressed on recombinant *Lactococcus lactis*, which conferred effective mucosal and systemic immune responses [106].

## 8. Challenges for Recombinant VLPs as Influenza Vaccines

Heterogenous composition is one of the challenges associated with the use of influenza VLPs as a vaccine candidate. VLPs produced by using baculovirus expression system contain live baculovirus vector as impurity. Moreover, considerable fraction of HA protein is bound to viral envelope of baculovirus [107]. Therefore, it is difficult to distinguish whether the HA activity is associated with influenza VLP or with baculovirus-displayed HA in the unpurified samples during vaccine manufacturing, which also causes difficulty for HA quantification. VLPs produced from mammalian expression system do not contain baculovirus contamination but they contain extracellular particles and produce low yields, with VLPs needing to be concentrated up to 200 times to reach measurable titers [72]. The current methods that are used for quantification are mostly applicable to concentrated and purified VLPs; however, better methods need to be developed to characterize the materials at the different stage of bioprocessing during the manufacturing process. Other potential challenges include the presence of endogenous rhabdovirus in standard Sf9 cell lines [108]. Although insect rhabdovirus is not associated with any human disease, the development of rhabdovirus-free cell line can resolve this potential safety concern [108,109].

To overcome manufacturing challenges, technological improvements to production of H7N9 VLPs in baculovirus-Sf9 insect cell cultures have been reported [110,111,112]. The yield and quality of influenza VLPs produced in insect cells could be improved by inhibiting cytopathic effects of the protein M2 [62]. The ion channel activity of M2 induces significant cytopathic effects in Sf9 insect cells. These effects include altered Sf9 cell morphology and reduced baculovirus replication, resulting in impaired influenza protein expression and thus VLP production. The use of the M2 inhibitor amantadine indeed improved Sf9 cellular expression not only of M2 (∼three-fold), but also of HA (∼seven-fold) and of matrix protein M1 (∼three-fold) when co-expressed to produce influenza VLPs. This increased cellular expression of all three influenza proteins further led to ∼two-fold greater VLP yield. The quality of the resulting influenza VLPs was significantly improved, as demonstrated by the ∼two-fold, ∼50-fold, and ∼two-fold increase in the antigen density to approximately 53 HA, 48 M1, and 156 M2 per influenza VLP, respectively [62].

For purification of influenza VLPs, a nitrocellulose membrane-based filtration system [113] and a cascade of ultrafiltration and diafiltration steps, followed by a sterile filtration step [109,114] were used successfully. The bioprocessing of influenza VLPs was recently reviewed [115]. Recently, a fast chromatography step purification method was developed for chimeric HIV-1 Gag influenza-HA VLPs produced in *Tnms*42 insect cells using the baculovirus insect cell expression vector system [116]. The absence of the baculovirus capsid protein p39 in the product fraction was confirmed by HPLC-MS. The process is simple and includes only a few handling steps, which has promising characteristics to become a platform for purification of these types of VLPs [116].

Regarding the detection and quality assessment of influenza vaccines, several methodologies are being used. These include development of standards for potency determination [117]. A high-resolution LC-MS method allows the quantitation of both HA and NA protein concentrations in influenza VLP vaccine candidates [118].

## 9. Conclusions

Recombinant VLPs were developed with the purpose of rapidly preparing influenza vaccines including against pandemic threat viruses such as H7N9 virus. Multiple designs of VLPs have been evaluated including the use of multivalent VLPs, multi-subtype mosaic VLP, and rationally designed VLPs containing conserved influenza virus epitopes. The use of highly conserved epitopes remains a promising approach for the improved design of highly immunogenic VLPs. Blended VLPs have been used as a classic approach to broaden immune coverage. Multi-subtype mosaic VLPs potentially combine the conserved epitope and blended approaches, as VLPs contain both subtype-specific head epitopes and the conserved stem epitopes. In addition to VLPs, other novel technologies have been used to develop improved influenza vaccines including DNA vaccines, viral vectors, advanced adjuvants, and other approaches. Potentially, the combination of various technologies can prove beneficial for the development of next-generation influenza vaccines including H7N9.

## Figures and Tables

**Table 1:** WHO-recommended vaccine strains for H7N9 virus (adapted from [21]).

Vaccine Prototype Strain	Vaccine Candidate	Examples
A/Guangdong/17SF003/2016	*Wild Type Virus*	
	Reverse genetics	CBER-RG7C, IDCDC-RG56N, CBER-RG7D, NIBRG-375
A/Hong Kong/125/2017	*Wild type virus*	
	Reverse genetics	IDCDC-RG56B
A/Shanghai/2/2013	*Wild type virus*	
	Reverse genetics	IDCDC-RG32A, IDCDC-RG32A.3, NIBRG-267, CBER-RG4A
A/Anhui/1/2013	*Wild type virus*	
	Reverse genetics	NIBRG-268, NIIDRG-10.1, IDCDC-RG33A, SJ005

**Table 2:** Examples of virus-like particle (VLP) vaccines against H7N9 influenza.

VLP Antigen	Expression System	Vaccination			Protection from Intranasal Challenge	References
		Animals	Dose	Route		
HA	High Five insect cells	mice	10 µg protein plus 10 µg poly(I·C) in PBS	IM	100 mLD_50_	[37]
HA	Insect Sf9	mice ferrets	5 µg 15 µg	IN IM	10 mLD_50_ 10^6^ PFU	[38]
HA	Insect Sf9	ferrets	15 µg	SC	10^7^ PFU	[31]
HA	Plants	mice ferrets	3 µg 15 µg	IM IM	100 mLD_50_ 10^6^ TCID_50_	[39]
HA, M1	Insect *Trichoplusia ni* cell line High Five (BTI-TN-5B1-4)	mice	0.03, 0.3, 3 µg	IM	fully protected against 100 mLD_50_	[40]
HA, M1	Insect Sf9	mice	1.5 µg	IN	10 mLD_50_	[41]
HA, NA, M1	Insect Sf9	mice	6 µg	IM	4.4X10^3^ TCID_50_ PFU	[42]
HA, NA, M1	HEK293T	mice	40 µg total protein	IM	NA	[43]
HA, NA, M1	Insect Sf9	mice	3 µg	SC	10^6^ TCID_50_	[44]
HA, NA, M1	Insect Sf9	mice chickens	10 µg	IM	NA	[45]
HA, NA, M1	Insect Sf9	mice	40 µg	IM, IN		[46]
HA, NA, BIV Gag	Insect Sf9	chickens	1536 HA units per dose of VLPs	SC	10^6^ EID_50_	[47]
HA/M1, M2/NA, BAFF-HA_tm_/M1, APRILHA_tm_/M1	Insect Sf9	mice	0.5 μg HA content	IM	10 mLD_50_ of NIBRG-14 vaccine strain	[48]
Combined HA and M2e5x (Universal)	Insect Sf9	mice	5 µg HA + 10 µg M2e5x VLPs	IM	7.5 x 10^2^ PFU = 5 mLD_50_	[49]

mLD_50_, mouse median lethal dose.
